# Functional Foods and Nutraceuticals in Irritable Bowel Syndrome

**DOI:** 10.3390/jcm14061830

**Published:** 2025-03-08

**Authors:** Giovanni Marasco, Cesare Cremon, Daniele Salvi, David Meacci, Elton Dajti, Luigi Colecchia, Maria Raffaella Barbaro, Vincenzo Stanghellini, Giovanni Barbara

**Affiliations:** 1 IRCCS Azienda Ospedaliero Universitaria di Bologna, 40138 Bologna, Italy; giovanni.marasco4@unibo.it (G.M.); daniele.salvi@poliambulanza.it (D.S.); luigi.colecchia@studio.unibo.it (L.C.); maria.barbaro2@unibo.it (M.R.B.); 2 Department of Medical and Surgical Sciences, University of Bologna, 40126 Bologna, Italy; v.stanghellini@unibo.it; 3 Department of Gastroenterology and Endoscopy, Fondazione Poliambulanza Istituto Ospedaliero, 25124 Brescia, Italy

**Keywords:** irritable bowel syndrome, disorders of gut–brain interaction, probiotics, essential oils

## Abstract

Irritable bowel syndrome (IBS) is a common disorder of gut–brain interaction, with a multifactorial pathophysiology involving gut–brain axis dysregulation, visceral hypersensitivity, microbiota imbalance, and immune dysfunction. Traditional IBS management emphasizes dietary modifications and pharmacologic therapies. However, increasing attention has been directed toward functional foods, nutraceuticals, and herbal remedies due to their potential to target IBS pathophysiological mechanisms with favorable safety profiles. This clinical review explores the role of these adjunctive therapies, evaluating evidence from preclinical and clinical studies. Functional foods such as kiwifruit, prunes, and rye bread demonstrate benefits in bowel habit regulation through fiber content and microbiota modulation. Nutraceuticals like peppermint oil, palmitoylethanolamide, and herbal mixtures exhibit anti-inflammatory, antispasmodic, and analgesic effects. Prebiotics provide substrate-driven microbiota changes, although dosage is key, as given their fermentative properties, when used at high dosages, they can exacerbate symptoms in some individuals. Probiotics and postbiotics offer microbiota-based interventions with promising symptom relief in IBS subtypes, although factors for personalized treatment still need to be further elucidated. These strategies highlight a paradigm shift in IBS management, integrating diet-based therapies with evolving nutraceutical options to improve patient outcomes. Despite promising findings, challenges in standardizing definitions, mechanisms, and safety profiles still remain. Rigorous, large-scale trials to validate the therapeutic potential of these interventions are needed, to enhance the benefits of these compounds with an individualized treatment approach.

## 1. Introduction

Irritable bowel syndrome (IBS) is a chronic gastrointestinal disorder characterized by recurrent abdominal pain associated with altered bowel habits in the absence of detectable structural or biochemical abnormalities [1,2]. IBS is classified into four subtypes based on predominant symptoms: with diarrhea (IBS-D), with constipation (IBS-C), mixed (IBS-M), and unclassified (IBS-U) [1]. The global prevalence of IBS is approximately 4.1% according to the Rome Foundation Global Epidemiology Study. IBS-C and IBS-M each account for about one-third of all IBS diagnoses, followed by IBS-D (28.7%) and IBS-U (6.5%). Regional variations range from 7.1% in Latin America to 1.8% in Southeast Asia [2]. Women and individuals under 50 years of age are more affected, and IBS contributes significantly to a diminished quality of life and increased healthcare costs globally [3]. The pathophysiology of IBS is complex and multifactorial, involving dysregulation of the gut–brain axis, visceral hypersensitivity, altered gut motility, and disruptions in the gut microbiome [4]. Visceral hypersensitivity, including a reduced threshold to the perception of luminal stimuli and the dysregulation of central processing, are key contributors to abdominal pain. Dysbiosis, characterized by reduced microbial diversity and an imbalance in gut microbial populations, may exacerbate symptoms through increased fermentation, impaired short-chain fatty acid (SCFA) production, and immune activation [5,6,7]. Several studies agree in showing increased Firmicutes and decreased Bacteroidetes, although less clear data are available at lower taxonomic levels, with Enterobacteriaceae, Lactobacillaceae, and Bacteroides being associated with harmful effects and bacteria of the Clostridiales family with protective roles [7]. Low-grade mucosal inflammation and intestinal barrier dysfunction further perpetuate symptoms by enhancing immune-mediated sensitization and intestinal permeability [5,6,7]. Neuroendocrine signaling involving serotonin, bile acids, and microbial metabolites also plays a critical role in modulating gut motility and secretion.

**Figure 1:**
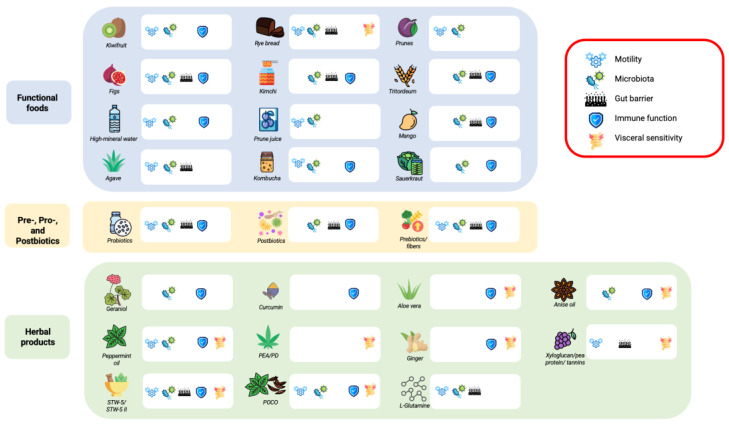
Main mechanisms of action of functional foods, herbal medicine, and other nutraceuticals in IBS. Each icon next to the medicinal products indicates which of the five main pathophysiological mechanisms of IBS the product targets.

First-line therapies for IBS include dietary interventions, such as the consumption of a low fermentable oligosaccharide, disaccharide, monosaccharide, and polyol (FODMAP) diet, fibers, and lifestyle changes, which have shown efficacy in reducing symptoms and improving quality of life [8,9,10,11,12,13,14,15,16,17]. Data on long-term effects of a low-FODMAP diet are limited, raising concerns about the potential nutritional deficits, gut microbiota alterations, and social acceptability of this therapeutic option [18]. Recently, interest has grown in adjunctive therapies, including functional foods, herbal medicines, and nutraceuticals [19]. These interventions act at various levels in IBS pathophysiology. Functional foods are defined as foods that provide health benefits beyond basic nutrition [20], such as fermented products (e.g., yogurt or kefir) and high-fiber foods, which target the gut microbiota by promoting microbial diversity and SCFA production, improving gut barrier function and immune modulation. Nutraceuticals are bioactive compounds derived from food, such as prebiotics, probiotics, and postbiotics, that exert medical or health benefits [21]. Herbal medicines include plant-derived compounds with pharmacological activity. Among herbal products, peppermint oil is a well-established treatment included in IBS guidelines due to its antispasmodic effect on intestinal smooth muscle, which reduces abdominal pain and discomfort [22]. However, despite promising evidence, variability in the efficacy, mechanisms of action, and safety profiles of these interventions presents challenges in routine clinical practice. We herein reviewed (Figure 1) the available evidence regarding the use of functional foods, nutraceuticals, and herbal medicines in IBS management in order to optimize personalized treatment approaches.

## 2. Functional Foods

Functional foods are a category of food that is still not well defined, although the Food and Agriculture Organization of the United Nations describes them as food that provides a health benefit beyond basic nutrition, demonstrating specific health or medical benefits, including the prevention and treatment of disease [20,23]. Despite the lack of a clear definition, functional foods have been used frequently as a therapeutic approach for IBS.

### 2.1. Fruit

#### 2.1.1. Kiwifruit

Several studies have investigated fruits in treating IBS-C and functional constipation (FC).

Kiwifruit contains 3% dietary fiber, which has a moderate water-holding capacity, and is rich in antioxidants, anti-inflammatory phytochemicals, and enzymes. Among enzymes, actinidin promotes gastric emptying and increases gut motility [24,25]. A 6-week diet enriched in kiwifruit in rats revealed the upregulation of genes involved in colonic barrier function (e.g., Mucin 2, Mucin 3, and Toll-Like Receptor 4), enhanced short-chain fatty acid (SCFA) production, and increased Lachnospiraceae taxa, which are beneficial for colonic health [26]. Similarly, kiwifruit consumption in healthy individuals has been associated with an increased abundance of protective bacteria, such as Bifidobacteria and Lactobacilli, in fecal samples [27]. Based on these properties, the consumption of two peeled kiwifruits per day is advisable for constipated patients [28,29]. Kiwifruit ingestion has been shown to improve stool consistency compared to the baseline in combined constipated patients (CC), IBS-C patients, and health controls, as measured using the Bristol stool form scale (BSFS) (combined constipation group +0.60, *p* < 0.001; IBS-C +0.71, *p* < 0.001). Additionally, stool frequency, as indicated by the number of complete bowel movements (CBMs) per week, improved in both FC patients (+1.53 CBMs/week, *p* < 0.001), IBS-C patients (+1.73 CBMs/week, *p* = 0.003), and health controls (+1.19 CBMs/week, *p* = 0.0022) compared with the baseline [30]. A recent meta-analysis confirmed that kiwifruit consumption is associated with higher bowel movements per week (BMs/week) compared to psyllium, another natural soluble fiber (FC +0.36 BMs/week, *p* < 0.001) [31]. Furthermore, kiwifruit is associated with lower gastrointestinal symptom rating scale (GSRS) scores for both constipation (FC −0.66 vs. −0.26, *p* = 0.03; CC −0.77 vs. −0.48, *p* = 0.02) and indigestion (FC −0.22 vs. −0.07, *p* = 0.04; CC −0.31 vs. −0.01, *p* = 0.005) when compared to psyllium [30]. Notably, these positive effects in constipated patients occur only with the whole fresh fruits but were not clearly confirmed with its supplements or dried powders [32,33]. This evidence underscores the great potential of kiwifruit as a functional food due to the complexity of its nutrient composition and food matrix. Furthermore, kiwifruit has shown very low adverse event rates, confirming the safety of this food in IBS treatment [28].

#### 2.1.2. Mango, Prune, and Fig

Other fruits investigated as potentially useful functional foods for constipation treatment include mangos, prunes, and figs.

Mangos are a fruit rich in fibers and polyphenols, which exert antibacterial and prebiotic functions. Preclinical studies demonstrated that mango consumption inhibits harmful bacteria, such as *Staphylococcus aureus*, *Escherichia coli*, and *Candida albicans*, while promoting beneficial microbes, including Lactobacillus, Bifidobacterium, Dorea, and Lactococcus [34]. In clinical studies, mango consumption (300 g per day) has shown to soften stools more than psyllium intake, as well as increase the production of short-chain fatty acids (SCFAs), in particular valeric acid, which exerts an anti-inflammatory effect in the intestines [35].

Prunes are a well-known functional food commonly recommended for constipated patients. Their high content of fibers and sorbitol, which have osmotic laxative properties, make them beneficial in IBS-C treatment [36]. Prune consumption is associated with an increase in beneficial bacterial populations, such as Bifidobacteria [37]. Twelve prunes per day were shown to be as effective as kiwifruits and psyllium in increasing stool frequency [28] after a 4-week period (+2.7 vs. +1.7 CBMs from the baseline; *p* < 0.01) and in increasing stool weight (+32.8 g/day vs. −0.8 g/day, *p* = 0.026) [37]. However, compared to kiwifruit consumption, prune intake has been associated with a higher incidence of adverse effects, likely due to their high fermentable potential, such as abdominal pain and bloating. Moreover, due to the high amount of sugar and calories, excessive consumption may contribute to unintended weight gain and elevated blood sugar levels, particularly concerning diabetic and overweight patients [28].

Figs are rich in phytonutrients and polyphenols, which exert antioxidant, anti-inflammatory, and immunomodulatory properties [38]. In a preclinical study in an ulcerative colitis-induced rat model, a fig extract showed protective effects against epithelial and glandular destruction, probably due to its antioxidant properties [39]. Figs’ efficacy in IBS-C and FC has been investigated in several RCT studies. Fig consumption significantly reduced the colonic transit time (38.7 ± 20.3 h vs. 46.7 ± 16.3 h, *p* = 0.045) and improved stool consistency (3.0 ± 0.8 vs. 2.8 ± 0.8 BSFS, *p* = 0.024) [40] while also alleviating IBS symptoms, including reduced pain frequency (−7.36 ± 13.02 vs. −1.69 ± 13.22, *p* = 0.04) and decreased severity of distention, as evidenced by a reduction in the IBS Symptom Severity Scale (IBS-SSS) compared with the placebo [41]. No significant adverse events from fig consumption were observed in studies involving constipated patients, as confirmed by a recent meta-analysis on the effects of various foods on CC [31].

### 2.2. Cereals

#### 2.2.1. Rye Bread

Recent insights have also emerged regarding cereals as potential functional foods in the treatment of IBS and FC. Rye bread, traditionally rich in fibers, may help alleviate symptoms in constipated patients. Two RCTs compared rye bread (seven slices per day) and probiotics with white bread in FC patients over a 3-week period [42,43]. In both studies, the rye bread group showed softer stools (60% vs. 27.3% subjects with softer stools, *p* = 0.037) and a reduced colonic transit time (42.5 vs. 65.5 h, *p* = 0.045) compared with the white bread group. However, rye bread was associated with a worsening in abdominal symptoms, such as bloating and flatulence [mean difference 1.6, 95% confidence interval (95% CI) 0.7 to 2.4; *p* < 0.001] likely due to its high FODMAP content. A pilot study by Pirkola et al. [44] suggested that modified low-FODMAP rye bread ingestion resulted in less abdominal distention (3.3 vs. 4.2 VAS measurement; *p* = 0.063) and colonic fermentation (6300 H ppm vs. 10,635 H ppm, *p* = 0.028), as measured via hydrogen excretion. These findings suggest that a low-FODMAP diet with rye bread in IBS-C patients may offer the positive effects in bowel habits without the adverse effects previously reported.

#### 2.2.2. Tritordeum

Tritordeum is a new cereal derived from wild barley and durum wheat crossbreeding [45]. Compared to wheat, it shows higher levels of antioxidant compounds, fibers, and proteins and lower levels of carbohydrates, fructans, and gliadin. In vitro studies showed that tritordeum fermentation increases in the Bifidobacterium population, increases the SCFA (butyrate and acetate) concentration, and improves intestinal barrier integrity [46]. Additionally, tritordeum assumption exerts anti-inflammatory properties, inducing IL-10 expression [46]. Two different studies carried out by the same research group showed that tritordeum-based foods can reduce symptoms in IBS-D patients. In one study, 12 weeks of tritordeum-based consumption was as effective as the low-FODMAP diet in improving their quality of life (QoL) and gastrointestinal symptoms, as evaluated by a reduction in the IBS-SSS total score (−130.5, 95% CI: −74.9 to −189.4, *p* < 0.0001) [47]. Additionally, a tritordeum-based diet improved the IBS Quality of Life (IBS-QoL) total score (from 68.54 ± 3.12 to 85.77 ± 1.97, *p* < 0.0001) and some subcategories of the 36-Short Form Survey, such as physical function, body pain, general health, vitality, and mental health [48].

#### 2.2.3. Agave

Agave is a food rich in fructans, with glycosidic linear β (2 → 1) linkages acting as prebiotics, and has demonstrated beneficial effects for human and gastrointestinal health, being associated with increased populations of Bifidobacteria and Lactobacilli [49]. Animal studies have correlated agave fructan intake with increased SCFA levels and higher populations of *Lactobacillus casei*, *L. paracasei*, and *L. rhamnosus bacteria*, exerting beneficial effects on health [50,51]. A recent RCT showed that its use in a jelly form in an IBS-C group led to significant reduced constipation (27.8% vs. 50.0% constipated patients after treatment) and an increased number of weekly bowel movements compared to the placebo. Agave fructan intake was also correlated with an improvement in QoL, measured as an increased IBS-QoL score after treatment (from 61.47 ± 5.84 to 76.06 ± 4.24). Furthermore, anxiety levels also showed a statistically significant reduction from the baseline after 30 days of consumption [52].

#### 2.2.4. Fermented Foods

Fermented foods, which are naturally rich in probiotics, such as kimchi and sauerkraut, have been associated with positive gut microbiota modulation and benefits ranging from the anti-atherosclerotic to anti-inflammatory effects [53]. A study conducted on obese murine models demonstrated that kimchi consumption could modulate the gut microbiota in mice [54]. This finding was later confirmed in human studies, where kimchi was shown to increase the abundance of short-chain fatty acid (SCFA)-producing bacteria, such as Faecalibacterium and Roseburia [55]. SCFAs produced by these bacteria, such as butyrate, are routinely used as postbiotics to alleviate IBS symptoms. Similarly, sauerkraut intake has been associated with an upregulation of antioxidant and detoxifying enzymes like Glutathione S-transferase (GST) and NAD(P)H:quinone oxidoreductase 1 (NQO1) in Wistar rats [56]. Two small studies evaluated the effects of these foods in IBS patients. A 12-week intake of kimchi was associated with reduced gastrointestinal symptoms, such as abdominal pain (from 2.4 ± 0.6 to 1.4 ± 0.5 on the Likert scale, *p* < 0.001) and bloating (from 2.6 ± 0.7 to 1.3 ± 0.5 on the Likert scale, *p* < 0.001), and a decrease in pro-inflammatory serum markers, such as TNF-α (from 6.5 ± 2.3 pg/mL to 3.3 ± 2.7 pg/mL, *p* < 0.001), compared to placebo [57]. Similarly, a Norwegian study showed that a 6-week sauerkraut supplement led to reduced IBS symptoms, as measured by a lowered IBS-SSS score (−57.0 ± 16.92, *p* = 0.003) and an increased level of Lactobacilli in gut microbiota from fecal samples (from 0% to 42% positivity, *p* = 0.047) [58].

### 2.3. Drinks

#### High-Mineral Water

High-mineral water may exert a positive effect in IBS-C patients. Compared with low-mineral water, high-mineral water contains more magnesium sulphate. This is a well-known mineral for its osmotic properties that can reduce the colonic transit time and soften stools [59]. Numerous studies have shown that 0.5–1.0 L/day intake of high-mineral water is more effective than low-mineral water in increasing BMs/week in FC patients (2.02 ± 2.22 BMs/week vs. 0.88 ± 1.67; *p* = 0.005) [60,61]. It is reasonable to speculate that a similar intake of high-magnesium water in IBS-C patients may lead to comparable improvements in constipation-related symptoms and might be associated with a reduction in stool consistency. Alternatively, magnesium oxide supplementation could achieve the same effects by softening stool consistency and increasing stool frequency [62]. The beneficial properties of magnesium are not limited to colonic motility. A study conducted on murine models with ulcerative colitis showed that a high-magnesium diet reduced disease activity (DAI) compared to a low-magnesium diet. The same study demonstrated that magnesium supplementation increased microbiota α-diversity in murine models of ulcerative colitis, enhanced Bifidobacterium populations, and reduced Enterobacteriaceae levels [63]. However, magnesium supplementation might affect drug absorption and should be avoided in elderly patients or those with renal failure [59].

### 2.4. Prune Juice

Prune juice, another potential drink for improving IBS-C symptoms, is rich in sorbitol, polyphenols, and pectin. Studies have shown that prune juice shapes the gut microbiota in obese rats, with increased levels of Turicibacter, Faecalibacterium, and Lactobacillus populations compared to controls [64]. In FC patients, this drink (54 g/die) was shown to be more effective than a placebo in reducing constipation (−2.24 ± 1.41 mean reduction in constipation GSRS item vs. −1.43 ± 1.56, *p* = 0.024) and softening stools (−2.31 ± 1.30 mean reduction in hard stool GSRS item vs. −1.33 ± 1.71, *p* = 0.009) [65]. On the other hand, prune juice might lead to some collateral effects, such as flatulence and diarrhea [66].

### 2.5. Kombucha

Kombucha has recently gained popularity among functional drinks. This sparkling fermented sweetened tea is rich in probiotics and contains numerous phytonutrients with anti-inflammatory and antioxidant properties [67]. In vitro studies have shown that kombucha has antimicrobial properties, inhibiting the growth of pathogenic bacteria such as *H. pylori*, *E. coli*, *S. typhimurium*, and *C. jejuni* [68]. In mice with non-alcoholic fatty liver disease (NAFLD), kombucha supplementation increased the abundance of Lactobacilli, with known anti-inflammatory and protective effects [69]. A small open-label RCT study explored kombucha enriched with inulin and vitamins in improving IBS-C symptoms. The kombucha group, which consumed 220 mL/day for 2 weeks, showed an increased stool frequency (from 0.60 ± 0.31 to 0.85 ± 0.19 times/d; *p* = 0.004), an improved BSFS consistency (4.4 ± 1.0 vs. 3.4 ± 1.2; *p* = 0.001), and a reduction in the sensation of incomplete bowel emptying (from 1.88 ± 0.78 to 1.41 ± 0.78 points on the Likert scale, *p* = 0.015) compared to the control group, which received only water [70].

In conclusion, although functional foods are supported by an abundance of literature suggesting their potential benefits on gut health and potential efficacy in the modulation of sensorimotor function, gut microbiota, and the epithelial barrier, evidence on functional foods as a therapeutic approach for IBS, in some instances, remains limited. However, given their biological potential and low production costs, further well-conducted studies are warranted to investigate their efficacy in the management of IBS symptoms.

## 3. Herbal Products and Other Nutraceuticals

Up to 36% of patients with DGBI report the use of nutraceuticals and herbal medicines, which are now gaining considerable attention as complementary or alternative therapies for IBS [19]. Herbal extracts offer a promising approach given their favorable safety profiles and potential to target multiple pathophysiological mechanisms underlying IBS. Although emerging clinical evidence suggests efficacy, further well-designed, rigorous trials are essential to confirm their role in IBS management [71]. In this section, we examine the major compounds currently available, highlighting the evidence from key studies.

### 3.1. Essential Oils

Peppermint oil, primarily composed of L-menthol, has been investigated in preclinical studies for its various mechanisms of action. These include calcium channel antagonism, facilitating gastrointestinal smooth muscle relaxation, the modulation of transient receptor potential (TRP) channels implicated in visceral nociception, kappa opioid receptor agonism, and notable anti-inflammatory and anti-infective properties [72,73,74,75].

Currently, two formulations of peppermint oil are commercially available: ileo-colonic and small-intestinal-release compounds. However, direct comparisons between these formulations remain limited and controversial, with one study reporting no statistically significant differences in abdominal pain response or overall symptom relief between either formulation and the placebo. Despite this, the small-intestinal formulation demonstrated significantly greater reductions in abdominal pain, discomfort, and overall IBS symptom severity than the placebo [76].

A recent meta-analysis of 10 randomized controlled trials (RCTs)—8 of which utilized a small-intestinal-release formulation—reported a relative risk of 0.65 (95% CI 0.47–0.88) for persistent global IBS symptoms or abdominal pain with peppermint oil compared with the placebo, yielding a number needed to treat (NNT) of 4. However, moderate-to-high heterogeneity was observed across studies, and only two trials employed the updated Rome IV criteria for diagnosing IBS. Gastroesophageal reflux is one of the most frequently reported adverse events associated with peppermint oil, with a relative risk of 1.67 (95% CI 1.18–2.38) [77]. Furthermore, an economic evaluation has identified small-intestinal-release peppermint oil as a cost-effective treatment from both societal and healthcare perspectives [78]. As a result, Western guidelines currently recommend its use as an antispasmodic agent in the management of IBS [8,9,10,11,12].

Peppermint oil can be combined with other essential oils to enhance its activity and obtain a synergic effect on gastrointestinal symptoms, such as in the combined formulations peppermint oil and caraway oil (POCO) and STW-5.

A peppermint oil and caraway oil combination, which exploits the anti-foaming proprieties of caraway oil, is suggested by Asian guidelines mainly for the treatment of fullness and bloating in functional dyspepsia (FD) [79]. However, in clinical and preclinical models, evidence exists of the reversal of visceral hypersensitivity in rats, which coincides with microbiome and mycobiome modulation [80,81]. A systematic review with a meta-analysis including patients with overlapping IBS and FD showed a reduction in common symptoms of IBS, such as flatulence and diarrhea, therefore representing a promising option in this setting [82].

STW-5, a combination of extracts from nine plants—*Iberis amara* L., *Carum carvi* L., *Glycyrrhiza glabra* L., *Mentha piperita* L., *Melissa officinalis* L., *Matricaria chamomilla* L., *Chelidonium majus* L., *Silybum marianum* L., and *Angelica archangelica* L.—has been shown to modulate motility, visceral hypersensitivity, inflammation, permeability, and the gut microbiota, all of which are relevant in the pathogenesis of IBS [83]. In a large cohort of IBS patients, both STW-5 and STW-5 II, a reduced formulation containing six extracts, exert similar pathophysiological mechanisms and were proven to be significantly more effective than a placebo in reducing the total abdominal pain scores regardless of IBS subtype [84,85].

Anise oil demonstrated antimicrobial, relaxant, and anti-inflammatory effects in murine models [86]. In a 4-week trial with 120 IBS patients, 75.5% of those in the anise oil group reported being symptom-free (0–10 VAS) compared to 52.5% in the peppermint oil group and 35% with the placebo. However, it did not significantly improve diarrhea or constipation [87].

Geraniol, an acyclic monoterpene extracted from lemongrass, rose, and other aromatic plants, has demonstrated strong antimicrobial activity along with antioxidant and anti-inflammatory properties [88,89]. In a pilot study of IBS patients, geraniol was shown to reduce symptoms, as assessed using the VAS-IBS total score, and to modulate gut microbiota by increasing the relative abundances of Collinsella and Faecalibacterium [90]. In an RCT involving all IBS subtypes, the geraniol group showed a significantly higher rate of responders, defined as an IBS-SSS reduction of ≥50 points (52.0% vs. 16.7%), particularly in the IBS-M subtype. This was associated with a significant decrease in the genus Ruminococcaceae and an increase in Faecalibacterium following geraniol administration [91].

### 3.2. Curcumin

Curcuminoids, derived from the rhizomes of Curcuma longa (turmeric), primarily consist of the natural polyphenol curcumin, along with demethoxycurcumin, bisdemethoxycurcumin, and cyclocurcumin. Curcumin has been extensively studied in animal models for its anti-inflammatory and antioxidant properties [92].

Three studies [93,94,95], including 326 individuals with IBS irrespective of subtype, met the assessment of risk of bias and were included in a meta-analysis [96]. The findings suggested that curcumin exerted beneficial, although not statistically significant, effects on IBS symptoms [93]. However, one study did not apply the Rome criteria for IBS diagnosis, and in the two other studies, curcumin was administered as part of a multi-herbal combination, raising consistent concerns regarding the ability to quantify and determine whether the therapeutic benefits were attributable specifically to curcumin. Importantly, no serious adverse events were reported across the studies. Nonetheless, appropriate longitudinal data are required, particularly given the evidence from animal models that high doses of curcumin can alter the pharmacokinetics of warfarin and clopidogrel [97].

### 3.3. Ginger

Ginger (Zingiber officinale), well known for its anti-emetic and analgesic properties, has been evaluated in a pilot RCT involving patients with IBS. The study showed that 1 g of ginger reduced IBS symptoms, as measured by the IBS Symptom Severity Score (IBS-SSS), in 26.4% of participants, although the effect was not significantly different from the placebo [98]. In a separate trial, ginger and Brewer’s yeast (*Saccharomyces cerevisiae*) were evaluated for their efficacy in managing IBS with constipation, with the ginger group showing a significant reduction in abdominal distention and constipation symptoms compared to the placebo [99].

### 3.4. Palmitoylethanolamide and Polydatin

Palmitoylethanolamide (PEA) is an endogenous N-acylethanolamine and an endocannabinoid-like lipid mediator known to modulate nociception through the regulation of mast cell activity [100]. Lower plasma levels of PEA in patients with IBS have been significantly associated with more severe abdominal pain [101]. In a pilot RCT, Cremon et al. evaluated the effects of oral supplementation of PEA and polydatin (PD), a resveratrol glucoside with potential synergistic effects, in 54 IBS patients. Although PEA/PD did not alter mast cell counts, it significantly reduced abdominal pain severity compared to the placebo [102].

More recently, Di Nardo et al. demonstrated that the same PEA/PD formulation significantly reduced abdominal pain severity in pediatric IBS patients, with a more pronounced effect in achieving complete remission after 12 weeks, particularly in those with diarrhea-predominant IBS [103]. In both adult and pediatric populations, PEA/PD supplements were well tolerated, with no severe adverse events reported.

### 3.5. Glutamine

L-glutamine, an essential amino acid important for gastrointestinal cell regeneration, has been studied for its potential to restore intestinal permeability. In IBS-D patients, the upregulation of miR-29a has been shown to reduce glutamine synthetase expression in the small intestinal and colonic mucosa, as well as decrease Claudin-1 and nuclear factor-κB-repressing factor levels, resulting in increased permeability [104,105].

It was hypothesized that glutamine administration could reverse the increased gut permeability associated with the onset of post-infectious IBS [105]. In an RCT, glutamine supplementation resulted in a significant reduction of ≥50 points in the IBS-SSS (79.6% vs. 5.8%) and improved stool frequency and form compared to the placebo. Additionally, it restored intestinal permeability, as measured by the lactulose/mannitol ratio [106]. Although the results of the study were promising, they need to be confirmed by further studies.

### 3.6. Other Nutraceuticals

Aloe barbadensis Mill., commonly known as aloe vera, has been shown to possess anti-inflammatory as well as immunomodulatory proprieties in vitro [107,108]. Its role in IBS was investigated in an RCT involving 68 patients diagnosed with IBS, according to the Rome III criteria. While the primary endpoint was not reached, there was a trend toward significance in the proportion of responders in the aloe vera group compared to the placebo (55% vs. 31%, *p* = 0.09). Notably, the aloe vera group demonstrated significant reductions in pain severity, pain frequency, and bloating compared to the placebo [109]. Also, another RCT involving all IBS subtypes found no difference in the frequency of responders, defined as an IBS-SSS reduction ≥50, between the aloe and control groups. Nevertheless, an analysis of the fecal microbiota and metabolite compositions identified responders exclusively in the aloe group, revealing distinct fecal microbiota and metabolite profiles [110]. Recently, carcinogenic activity of aloe vera whole-leaf extract has been demonstrated in rats; therefore, the compound is currently classified as a possible human carcinogen (Group 2B), prompting a global warning on its use [111].

A medical device containing xyloglucan, pea protein, tannins extracted from grape seeds, and xylo-oligosaccharides has been studied in patients with IBS-D due to its ability to inhibit stress-induced visceral hypersensitivity and gut hyperpermeability in animal models [112]. In a double-blind crossover trial, it was demonstrated that the device effectively controlled diarrhea and improved abdominal pain, bloating, and quality of life compared to the placebo [113]. These findings were further supported by a long-term safety study, which showed sustained symptom improvement over a 6-month extension period in the absence of any severe adverse event [114].

In conclusion, herbal products and nutraceuticals show overall benefits in improving IBS symptoms. Peppermint oil has strong evidence supporting its use, earning a recommendation in European and American IBS guidelines [8,9,10,11,12]. Promising results are emerging for xyloglucan, pea protein, tannins, and xylo-oligosaccharides. However, other nutraceuticals show mixed results, limited by small sample sizes and trial design, underscoring the need for further research to confirm their therapeutic potential.

## 4. Prebiotics, Probiotics, and Postbiotics

### 4.1. Prebiotics

The International Scientific Association for Probiotics and Prebiotics (ISAPP) published, in 2017, a consensus definition of prebiotics, defined as “substrates that are selectively utilized by host microorganisms conferring a health benefit” [115]. This definition was recently reviewed and confirmed by an Expert Recommendation [116], emphasizing the importance of establishing a causal link between prebiotic-induced selective effects of the microbiota and health benefits.

Prebiotics include various fermentable carbohydrates, such as digestible oligosaccharides, fructans and galactans, including fructooligosaccharides (FOSs) and galactooligosaccharides (GOSs), inulin, oligofructose, lactulose, and breast milk oligosaccharides [115,116,117]. In addition, some fermentable fibers are also classed as prebiotics, including inulin-type fibers and galactooligosaccharides [118]. Prebiotic fibers are known for their rapid fermentative capacity and subsequent release of short-chain fatty acids (SCFAs), in particular acetate, but they also selectively stimulate the growth of a specific range of genera and/or species (Bifidobacterium and Lactobacillus) [118]. The physicochemical characteristics (solubility, viscosity, and fermentability) of dietary fibers might be relevant for the function of the gastrointestinal tract, contributing to stool output (frequency, consistency, and weight) and stimulating changes in microbial composition and metabolite production, including the production of SCFAs [118]. To date, systematic reviews and meta-analyses indicated that some fibers are beneficial in reducing IBS symptoms and improving stool frequency and consistency, although the results are inconsistent and probably limited to soluble fibers [118].

While prebiotics can offer benefits like SCFA production, pathogen inhibition, immune stimulation, and improved mental health, their impact on IBS is controversial [115,116,117]. This is because the fermentation of carbohydrates can lead to gas production, potentially exacerbating symptoms in individuals with DGBIs. In fact, some prebiotics belong to the group of FODMAPs, while others, like inulin and FOSs, contain fructose, which can cause bloating, flatulence, and abdominal pain [119,120]. In contrast, GOSs have shown potential for symptom improvement in IBS, although the highest beneficial effects were reached without exceeding 3.5 g/day [121,122,123]. Although the World Gastroenterology Organisation Global Guidelines suggest that some prebiotics, including short-chain FOSs and GOSs, may be beneficial in IBS [124], the impact of prebiotics on DGBIs requires further investigation, as the current understanding is not yet conclusive.

### 4.2. Probiotics

Based on the Food and Agriculture Organization/World Health Organization (FAO/WHO) definition and the ISAPP consensus, probiotics are actually defined as “live micro-organisms that, when administered in adequate amount, confer a benefit to the host” [125]. They may exert health effects through various mechanisms, including an impact on the mucosal immune system, interaction with commensal or pathogenic microbes, the production of metabolic end products (in particular, SCFAs), and chemical signaling with host cells [117,125]. On these bases, probiotics can improve the intestinal microbial ecosystem, strengthen the intestinal barrier, and modulate immune responses, making them particularly attractive for disorders in which the above-described pathophysiological mechanisms have been hypothesized, such as DGBIs in general and, in particular, IBS [6,7,117,125].

In this context, recent studies have explored how probiotics can benefit patients with IBS by modulating their gut microbiota and its metabolic pathways. A randomized, double-blind, crossover, placebo-controlled trial assessing the efficacy and the mechanism of action of Lactobacillus paracasei CNCM I-1572 in patients with IBS showed initial improvements in IBS symptoms, a significant reduction in the genus Ruminococcus, an increase in fecal SCFAs such as acetate and butyrate, and a reduction in the pro-inflammatory cytokine interleukin-15 [126]. These changes suggest that this probiotic can effectively modulate gut microbiota and reduce immune activation in patients with IBS [88]. In addition, Lactobacillus rhamnosus HN001 and Bifidobacterium longum BB536 were shown to modulate the gut microbiota composition in healthy controls, significantly reducing potentially harmful bacteria [127]. A formulation containing these probiotics improved IBS symptoms and restored intestinal permeability in 25 Rome IV IBS patients [128]. A recent RCT on Lacticaseibacillus paracasei CNCM I-1572 in non-constipated IBS patients identified Collinsella aerofaciens as a potential predictive marker for response to probiotic treatment. In particular, responders exhibited an increased abundance of this pathobiont, which could be reduced by probiotic intake [129]. These findings highlight the potential of probiotics to modulate gut microbiota and improve IBS symptoms. The identification of Collinsella aerofaciens as a predictive marker may help tailor probiotic treatments to individual patients, enhancing their efficacy and addressing the issue of non-responders. However, further research is needed to fully understand the mechanisms involved in gut microbiota modulation and to confirm these predictive markers.

Several meta-analyses evaluated the efficacy and safety of probiotics in IBS [130,131]. A systematic review including 82 trials and 10332 patients indicated that some combinations of probiotics or strains may be beneficial, although the evidence was low or very low [132]. In particular, for global IBS symptoms, the effectiveness was noted for Escherichia, Lactobacillus, *L. plantarum* 299 V, combination probiotics, and Bacillus strains [132]. For abdominal pain, benefits were observed with *S. cerevisiae* I-3856, Bifidobacterium, and various combination probiotics [132]. For abdominal bloating or distension, evidence supports the use of combination probiotics and Bacillus strains [132]. Finally, for IBS-D, combination probiotics and Lactobacillus strains showed improvement in global symptoms, while data for IBS-C are limited [129]. Notably, the relative risk of adverse events was not significantly different between probiotics and placebos in studies involving over 7000 patients [132]. Further research is needed to consistently confirm these findings.

Based on the available randomized control trials (RCTs) and meta-analyses, various guidelines and consensus statements from major Gastroenterological Associations or Societies across different regions provide different recommendations regarding the use of probiotics for managing IBS [8,9,10,11,12,13,14,15,16,17]. The American Gastroenterological Association (AGA) and American College of Gastroenterology (ACG) guidelines on IBS management suggest the use of probiotics only within the context of clinical trials, emphasizing the need for more robust evidence due to the low quality of existing studies [11,13,14,15]. In contrast, the Canadian, Indian, European, British, and Italian guidelines support the use of probiotics for IBS symptoms, although their recommendations are conditional and based on low or very low levels of evidence [8,9,10,12,15,16,17]. In addition, there is a consensus on the potential benefits of probiotics for IBS, since they may help improve the overall symptoms of IBS, abdominal pain, or diarrhea, but specific strains are not identified. In all cases, the recommendations are conditional due to the heterogeneity of trials regarding the strains and combinations used, study designs and endpoints, and potential publication bias. All guidelines agree that more studies are necessary to clarify the effectiveness and safety of probiotics in IBS management, highlighting the need for further research to establish and improve the quality of evidence.

### 4.3. Postbiotics

Postbiotics, as defined by the ISAPP, are “preparations of inanimate (non-viable) microorganisms and/or their cellular components that confers a health benefit to the host” [133]. Recent research has highlighted their potential in managing IBS. In particular, a multicenter, randomized, double-blind, placebo-controlled trial demonstrated that heat-inactivated Bifidobacterium bifidum MIMBb75 was effective and safe for improving symptoms in 443 patients with IBS, regardless of bowel habit. The active treatment achieved the composite primary endpoint, which included at least a 30% improvement in abdominal pain and a significant relief of global IBS symptoms in at least 50% of the treatment period [134]. Emerging evidence suggests that postbiotics may offer benefits for IBS patients, but more extensive and well-designed studies are necessary to confirm their efficacy and establish their use in clinical practice.

In conclusion, the overall role of prebiotics, probiotics, and postbiotics in managing IBS, although promising, is still controversial and requires further investigation and standardization.

## 5. Conclusions

Functional foods such as kiwifruit, prunes, and rye bread demonstrate benefits in bowel habit regulation through their fiber content and microbiota modulation; however, some limitations are given due to possible side effects, such as bloating and distension, and the amount of food that needs to be eaten to exert an effect. Moreover, although larger amounts of data are available in preclinical trials, only small clinical trials on humans have been conducted, highlighting the need to validate the current results with larger human trials. Nutraceuticals like peppermint oil, palmitoylethanolamide, and herbal mixtures exhibit anti-inflammatory, antispasmodic, and analgesic effects. Prebiotics provide substrate-driven microbiota changes, although their fermentative properties can exacerbate symptoms in some individuals. Probiotics and postbiotics offer microbiota-based interventions with promising symptom relief in IBS subtypes, albeit with varied efficacies and the need for strain-specific research. These strategies highlight a paradigm shift in IBS management, integrating diet-based therapies with evolving nutraceutical options. Despite promising findings, challenges in standardizing the definitions, mechanisms, and safety profiles still remain. Rigorous, large-scale trials to validate the therapeutic potential of these interventions are needed, with some of them being currently underway, to enhance the benefits of these compounds using an individualized treatment approach.

## Data Availability

The data presented in this study are openly available in Medline and Embase.
